# Receptor activator of nuclear factor-kappa B is enriched in CD9-positive extracellular vesicles released by osteoclasts

**DOI:** 10.20517/evcna.2023.38

**Published:** 2023-09-06

**Authors:** Shaobo Ruan, Wellington J. Rody Jr, Shivani S. Patel, Lina I. Hammadi, Macey L. Martin, Lorraine P. de Faria, George Daaboul, Leif S. Anderson, Mei He, Lexie Shannon Holliday

**Affiliations:** ^1^Department of Pharmaceutics, College of Pharmacy, Gainesville, FL 32610, USA.; ^2^Department of Orthodontics and Dentofacial Orthopedics, University of Pittsburgh, School of Dental Medicine, Pittsburgh, PA 15261, USA.; ^3^Department of Orthodontics, University of Florida College of Dentistry, Gainesville, FL 32610, USA.; ^4^Department of Biomaterials and Oral Biology, School of Dentistry, University of São Paulo, São Paulo - SP 05508-000, Brazil.; ^5^Unchained Labs, Pleasanton, CA 94588, USA.; ^6^Department of Anatomy & Cell Biology, University of Florida College of Medicine, Gainesville, FL 32610, USA.

**Keywords:** Exosomes, microvesicles, bone, remodeling, Single Particle Interferometric Reflectance Imaging Sensor, RANKL reverse signaling

## Abstract

**Aim:**

Receptor activator of nuclear factor-kappa B (RANK)-containing extracellular vesicles (EVs) bind RANK-Ligand (RANKL) on osteoblasts, and thereby simultaneously inhibit bone resorption and promote bone formation. Because of this, they are attractive candidates for therapeutic bone anabolic agents. Previously, RANK was detected in 1 in every 36 EVs from osteoclasts by immunogold electron microscopy. Here, we have sought to characterize the subpopulation of EVs from osteoclasts that contains RANK in more detail.

**Methods:**

The tetraspanins CD9 and CD81 were localized in osteoclasts by immunofluorescence. EVs were visualized by transmission electron microscopy. A Single Particle Interferometric Reflectance Imaging Sensor (SP-IRIS) and immunoaffinity isolations examined whether RANK is enriched in specific types of EVs.

**Results:**

Immunofluorescence showed CD9 was mostly on or near the plasma membrane of osteoclasts. In contrast, CD81 was localized deeper in the osteoclast’s cytosolic vesicular network. By interferometry, both CD9 and CD81 positive EVs from osteoclasts were small (56-83 nm in diameter), consistent with electron microscopy. The CD9 and CD81 EV populations were mostly distinct, and only 22% of the EVs contained both markers. RANK was detected by SP-IRIS in 2%-4% of the CD9-containing EVs, but not in CD81-positive EVs, from mature osteoclasts. Immunomagnetic isolation of CD9-containing EVs from conditioned media of osteoclasts removed most of the RANK. A trace amount of RANK was isolated with CD81.

**Conclusion:**

RANK was enriched in a subset of the CD9-positive EVs. The current study provides the first report of selective localization of RANK in subsets of EVs.

## INTRODUCTION

Receptor activator of nuclear factor-kappa B-ligand (RANKL), on osteoblasts or osteocytes, binds its receptor RANK on osteoclast precursors and osteoclasts, and this is required for osteoclast formation, osteoclast survival, and bone resorption^[1,2]^. Bone diseases, in particular osteoporosis and metastatic bone cancer, are major clinical problems that are associated with excess and/or improperly localized bone resorption by osteoclasts. Amgen developed a humanized monoclonal antibody directed against RANKL (denosumab), which blocks its interaction with RANK, which has become a major therapeutic agent for the treatment of osteoporosis (*Prolia*) and bone cancer (*Xgeva*)^[3-5]^.

Recently, *in vitro* evidence suggested that EVs-containing RANKL and EVs-containing RANK (RANK-EVs) are involved in bone remodeling^[6-10]^. Osteoblasts produce EVs containing RANKL that can stimulate osteoclast formation^[6,11]^. Osteoclasts produce RANK-EVs that can block RANKL binding to RANK to inhibit bone resorption^[8]^. In addition, when RANK-EVs bind RANKL on cells of the osteoblastic lineage, they stimulate them to differentiate towards bone formation through a RANKL-reverse signaling pathway^[12]^. Consistent with *in vitro* studies, it was demonstrated that RANKL-EVs stimulate osteoclasts *in vivo* in mice and zebrafish^[7,13]^. RANK-EVs regulate bone remodeling *in vivo* in mice by two mechanisms: they both reduce bone resorption and stimulate bone formation^[12]^. This identified RANK-containing EVs, or agents that mimicked their effect, as potentially attractive therapeutic agents^[12,14]^. These data suggest that the incorporation of RANK and RANKL into EVs provides new modes by which these proteins are involved in regulating bone remodeling^[9]^.

Only a small percentage of EVs released by osteoclasts contain RANK^[8]^. However, because of the high binding affinity between RANKL and RANK^[15]^, a small number of RANK-containing EVs could have important regulatory effects. It was recently directly confirmed that RANK in EVs binds RANKL with an affinity of from 1-10 × 10^-9^ M^[16]^. In principle, agents that could stimulate osteoclasts to release higher numbers of RANK-EVs during their normal life cycle should reduce bone resorption and stimulate bone formation in the same way as the addition of exogenous RANK-EVs or RANK-EV mimetics. Indeed, such a mechanism might avoid possible off-target effects by localizing the increased RANK-EV levels to the bone microenvironment being remodeled. Because the RANKL/RANK signaling network also plays an important role in communication between immune cells^[17,18]^, off-target effects are a real concern if RANK-EV levels are increased systemically. Unfortunately, nothing is known about the subset of EVs from osteoclasts that contain RANK, so there is no basis for rational strategies to stimulate RANK-EV production. In this study, we have made use of a Single Particle Interferometric Reflectance Imaging Sensor (SP-IRIS)^[19,20]^ and immunoaffinity isolations to analyze RANK-EVs in greater detail. We hypothesized that RANK would be localized to a specific subset of EVs that also contains particular marker proteins, and that this would reflect the protein trafficking pathway through which RANK is incorporated into EVs. By SP-IRIS and immunoaffinity isolation, we have tested this idea.

## EXPERIMENTAL PROCEDURES

### Reagents and antibodies

Minimum essential media, α modification (αMEM) and Dulbecco’s minimum essential media (dMEM) were procured from Sigma/Aldrich Chemical CO (St. Louis, MO). The Anti-RANK antibody (Cat # Orb6560) was from Biorbyt (Cambridge, UK) and used in Westerns. The anti-RANK antibody for SP-IRIS, R12-31 (Cat # 14-6612-82), was obtained from Thermo Fisher. Anti-CD9 antibody MM2/57 Millipore/Sigma (cat# CBL162, Millipore Sigma) and anti-CD81 SN206-01 (Catalog # MA5-32333, ThermoFisher) were used in Westerns, immunofluorescence, and for EV isolation using immunomagnetic beads. For SP-IRIS, the following antibodies were used: CD81 (JS-81, BD Biosciences Cat# 555675), and CD9 (HI9A, ThermoFisher Cat# 605-470) for capture. Secondary antibodies were obtained from Sigma-Aldrich (St. Louis, MO, Cat #s T7782; F4890) for immunofluorescence, or Thermofisher (Cat #s A16066; 65-6120) for blots. ExoQuick TC was acquired from Systems Biosciences (Mountain View, CA, Cat # EXOTC50A-1).

### Cell culture

Primary osteoclasts were from the marrow of the long bone of the hind legs obtained as described^[8]^. Mice (C57BL/6, Charles River) were sacrificed by cervical dislocation. The femora and tibia were removed, and marrow was flushed with α-MEM complete media (Sigma-Aldrich) plus 10% exosome-free fetal bovine serum (Systems Biosciences, EXO-FBSHI-250A-1) supplemented with 1% L-glutamine (Thermo Fisher Scientific), and 1% penicillin/streptomycin/am using a syringe with a 25-gauge needle. Cells were cultured in 100-cm dishes. They were seeded at a concentration of 1.5 × 10^6^ cells/mL. Media was supplemented with 5 ng/mL recombinant murine Macrophage-Colony Stimulating Factor [CSF-1] (Peprotech, ThermoFisher Cat # AF-315-02-10UG). Cells were allowed to grow for 24 h at 37 °C and 5% carbon dioxide. Nonadherent cells were removed, and 5.9 × 10^5^ cells/mL of adherent cells were placed in 24-well plates or at 2.1 × 10^6^ on 6-well plates. Cultures were stimulated with 5 ng/mL CSF-1 and 5 ng/mL soluble recombinant RANKL (sRANKL) produced in the lab as described previously^[21]^. Osteoclasts formed after 5 or 6 days with α-MEM with 10% exosome-free fetal bovine serum (System Biosciences) and CSF-1 and RANKL refreshed every 3 days. Conditioned media for osteoclasts was taken from days 3-6 of culture; precursor EVs were isolated from cells grown with CSF-1, but not RANKL for three days.

4T1 murine breast cancer cells (kind gift of Gary Sahagian, Tufts University, Boston, MA) were grown in 6-well plates indMEM plus 10% exosome-free fetal bovine serum (Systems Biosciences)^[22]^. Conditioned media was collected when cells were 50%-80% confluent^[23]^.

### Microscopy

Primary osteoclasts, osteoclast precursors, or 4T1 cells were fixed with 2% formaldehyde in PBS for 20 min and permeabilized with 0.5% Triton X-100 in PBS. Cells were grown in 24-well plates with Osteoclasts and pre-osteoclasts were then stained for tartrate-resistant acid phosphatase (TRAP) activity using Leukocyte acid phosphatase kit (Sigma-Aldrich catalog #386A). 4T1 cells were stained with Phalloidin-Tetramethylrhodamine B isothiocyanate (Sigma-Aldrich, Cat # P1951, 10 ng/mL) to detect microfilaments. Images were taken using a Nikon Diaphot phase contrast microscope (ELWD 0.3).

Primary osteoclasts were stained for CD9 and CD81. They were fixed in 2% formaldehyde in PBS for 20 min, washed with PBS two times for 10 min each, and blocked in blocking buffer [PBS with a pH of 7.4 plus 1% bovine serum albumin (BSA)] for 60 min. The cells were then probed with 200 μL of the mouse anti-CD9 (MM2/57, diluted 1:500), or anti-CD81 (SN206-01, diluted 1:500) at 4 °C overnight. Dilutions were done in blocking buffer. Then, the cells were washed two times in PBS for 10 min each, and secondary antibodies rhodamine-conjugated to mouse IgG (T7782, diluted 1:500) or fluorescein-conjugated anti-rabbit (F4890, 1:500) were incubated for 60 min at room temperature in the dark. The cells were rinsed two times in PBS for 10 min each, and fluorescence was detected by Nikon epifluorescence microscopy. ImageJ and/or Adobe Photoshop and Adobe Illustrator were used to prepare Figures.

### EV isolation

EVs were isolated under sterile conditions as described previously^[23]^ using ExoQuick TC material from conditioned media following the manufacturer’s instructions. The final pellet, containing EVs and ExoQuick, was diluted fivefold with PBS to solubilize the ExoQuick material. The samples were then spun at 200,000 × *g* for 2 h in an Airfuge (Beckman Coulter, Brea, CA, USA) and the pellets were collected.

### Transmission electron microscopy

EVs were prepared for electron microscopy as described previously^[8]^. EVs were isolated and fixed in 2% paraformaldehyde. The fixed EVs were placed on Parafilm, and Formvar-carbon-coated electron microscope (EM) grids were floated on the EVs for 20 min. Grids were washed in PBS, transferred to 1% glutaraldehyde for 5 min, and then washed 8 times in deionized water. EVs were then placed atop a drop of uranyl-oxalate solution, which was transferred to methyl cellulose-uranyl acetate on ice for 12 min. The grids were blotted dry with filter paper, and observed under a Hitachi (Tokyo, Japan) 7600 transmission electron microscope operated at 80 kV.

### Immunoblots

Proteins were separated by sodium dodecyl sulfate polyacrylamide gel electrophoresis on 4%-20% gels using the Mini-Protean system (BioRad). Gels were transferred to Immobilon membranes (Thermofisher Cat # 88518). The membranes were then probed with antibodies to RANK (Orb6560, diluted 1:1000), CD9 (MM2/57, diluted 1:1000), or CD81 (SN206-01, diluted 1:1000) and a horseradish peroxidase-conjugated secondary antibody (ThermoFisher Cat # 65-6120, diluted 1:10,000) was used to detect rabbit primary antibodies and a horseradish peroxidase-conjugated secondary antibody (ThermoFisher Cat # A16066, diluted 1:5000) were used to detect mouse primary antibodies. These were incubated in a chemiluminescent substrate (ThermoFisher, Super Signal West Pico, Cat # 34577), and blots were detected using a BioRad ChemiDoc MP (BioRad). The raw photographs and chemiluminescent data were minimally processed using brightness and contrast controllers equally over the whole blot in Adobe Photoshop for final figures.

### SP-IRIS

We used the ExoView R-100 (Nanoview Technologies) as our SP-IRIS to analyze EVs released by osteoclasts. ExoView analysis was performed utilizing a specific chip (Cat # EV-TETRA-C, Nanoview Technologies). This method combines single particle interferometric reflectance imaging sensing with antibody-based microchip capture and fluorescence detection to measure EV size and concentration, the presence of EV tetraspanins CD9 and CD81 and RANK, and their colocalization profile. Preliminary experiments established the appropriate dilutions for detection of EVs from conditioned media of osteoclast pre-osteoclasts and 4T1 cells. 35 μL of diluted cell supernatants were placed on microchip with separate wells pre-coated with the capture antibodies CD81 (clone Eat-2, mouse, Biolegend), CD9 (clone MZ3, mouse, Biolegend), and negative controls HIgG (clone HTK888, mouse, Biolegend) and RIgG (clone RTK2758, Biolegend). The microchips were incubated overnight, followed by three washing steps, blocking and incubation with a cocktail of fluorescent antibodies against RANK, CD81 and CD9, allowing for colocalization analysis of the three membrane proteins on single EVs. Probe antibodies were Alexa Fluor@488-conjugated CD9 (MM2/57), Alexa Fluor@555-conjugated CD81 (SN206-01), Alexa Fluor@647-conjugated RANK (R12-31). This was followed by two additional washes. The ExoView R100 reader and nScan 2.8.19 acquisition software (NanoView) were used for imaging microchips and data acquisition. The ExoView Analyzer 3.2 with thresholds set to 50-200 nm was used to perform data analysis. All measurements were done in triplicate.

### Immunomagnetic isolation

Anti-CD9 antibody MM2/57 or an anti-CD81 antibody SN206-01 were dialyzed into PBS and then coupled to Dynabeads M-270 Epoxy Beads (Cat # 65305, Thermo Fisher Scientific). All necessary reagents were provided in the Dynabeads Antibody Coupling Kit for magnetic bead isolation (Cat # 65305). The procedure was performed as described in the manual provided with the kit. Conditioned media dialyzed into 20 mM Tris (pH 7.4), 1 mM EDTA, 1 mM dithiothreitol, and protease inhibitors (F-buffer) were incubated for 2 h with 50-µL beads; the supernatants were collected; and the beads were washed thoroughly with F-buffer. Bead-bound material was eluted with 100 mM glycine sulfate (pH 2.3).

### Statistics

Groups were first analyzed by ANOVA, then if significant, by Student’s *t*-test. Statistics were performed using SPSS version 17.0 (IBM).

## RESULTS

### Location of CD9 and CD81 in osteoclasts

To make use of the ExoView SP-IRIS, it is necessary to select antibodies that bind to abundant surface receptors on the EVs to be studied. From our previous quantitative proteomic analysis of EVs from osteoclasts, we identified the tetraspanins CD9 and CD81 to be abundant EV markers in osteoclasts EVs^[24]^. The most abundant of the “standard” EV markers in osteoclast EVs was syntenin-1^[24,25]^; however, an appropriate capture antibody for SP-IRIS is not available. CD9 and CD81 were attractive because they have roles in osteoclast differentiation and function^[26-28]^.

**Figure 1:**
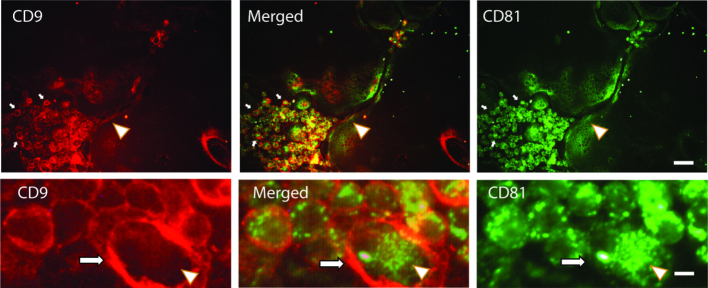
CD9 and CD81 have different localization patterns in osteoclasts and their precursors. Upper panels. CD9 (red, left panels) is concentrated in the plasma membrane area of unfused osteoclasts (small arrows) and of mature osteoclasts (arrowhead). CD81 (green, right panel) stains internally in unfused osteoclasts (small arrows) and in osteoclasts and many areas of cytosolic staining do not overlap. Merged image shows some overlap (yellow), but subsets of CD9 and CD81 do not co-localize. Bottom panels show a blowup of a region containing unfused osteoclasts, showing the differential distribution of CD9 and CD81 in more detail. Arrow indicates CD9 stained plasma membrane. Arrowhead indicates CD81 stained cytosol. Scale bar = 20 µm in top panels and 2.5 µm in bottom panels.

We performed an immunofluorescent analysis of these proteins in osteoclasts and found that CD9 and CD81 were mostly localized in different cellular regions [Figure 1]. The difference was most dramatic in early unfused osteoclasts, where CD9 was distinctly associated with the plasma membrane while CD81 was mostly in the cytosol. In osteoclasts, the labeling patterns of both CD9 and CD81 were less distinct, but they mostly were not co-localized. Higher magnification shows that the two are very well separated in unfused osteoclasts. These data suggested that CD9 and CD81 occupy different subcellular locations in cells of the osteoclast lineage and are candidates to emerge from different pathways of EV production.

### Validation of EV producing cells

**Figure 2:**
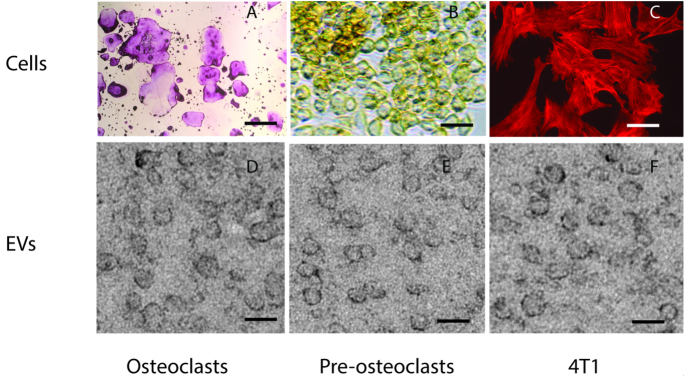
Extracellular vesicles isolated from osteoclasts, osteoclast precursors and 4T1 cancer cells have similar sizes and morphology. Top panels, (A) low magnification view of osteoclasts stained for tartrate-resistant acid phosphatase (TRAP) activity shows mostly multinuclear giant cells that stain pink indicating they are positive for TRAP; (B) Higher magnification view of osteoclast precursors, also stained for TRAP activity, shows no pink stain, only gold background staining; (C) Phalloidin-tagged with Texas Red demonstrates the 4T1 cancer cells; 4T1 cells do not contain TRAP. Scale bar in top panels is 200 µm for osteoclasts, and 10 µm for osteoclast precursors and 4T1 cells; Bottom panels show EVs isolated from (D) osteoclasts; (E) pre-osteoclasts and (F) 4T1 cells. Note that the EVs from the different cell types share similar size and morphology. Scale bars for bottom panels = 60 nm.

Osteoclasts or osteoclast precursors were grown in cell culture as described previously^[23]^. To ensure the quality of the cells from which the EVs were obtained, we fixed the cells and stained osteoclasts and osteoclast-precursors for tartrate-resistant acid phosphatase (TRAP) activity, a marker for osteoclasts. Phalloidin was used to label the microfilament cytoskeleton of 4T1 cells [Figure 2, top panels]. Osteoclast cultures contained many giant TRAP-positive multinuclear cells, as well as smaller TRAP-positive multinuclear and mononuclear cells [Figure 2A]. Osteoclast precursors displayed no TRAP activity and were mononuclear [Figure 2B]. 4T1 murine breast cancer cells, in which RANK is not detected, were used as a negative control in these studies [Figure 2C].

EVs were isolated from conditioned media from each type of culture [Figure 2D-F]. The EVs displayed a typical cup-like appearance. The sizes of EVs determined were osteoclasts (53 ± 12 nm *n* = 137), preosteoclasts (47 ± 13 nm, *n* = 64), and 4T1 cells (60 ± 16, *n* = 111). The sizes of the EVs from the different cells were not significantly different.

### Data from SP-IRIS

**Figure 3:**
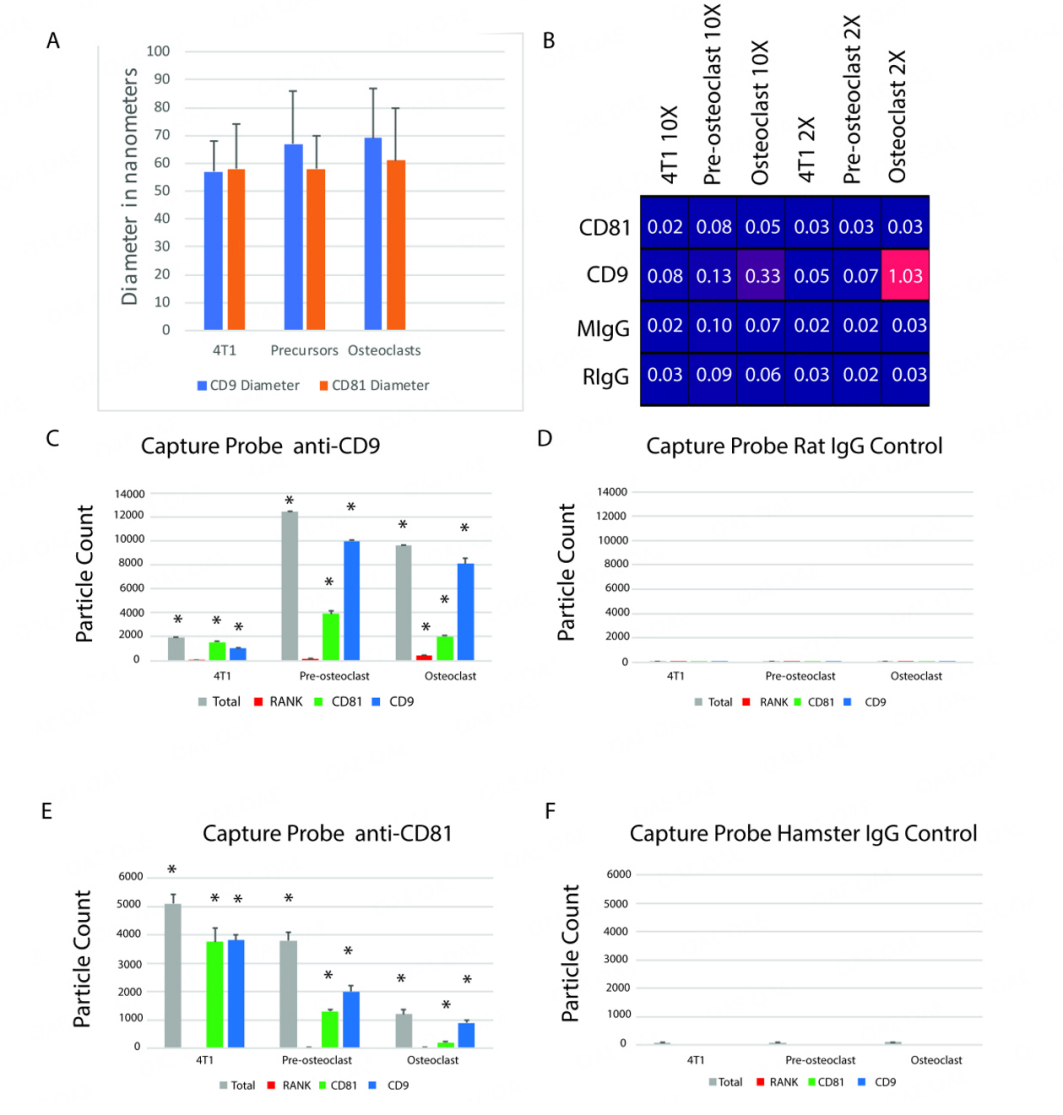
Characterization of CD9 and CD81 EVs by SP-IRIS. (A) Sizes measured by interferometry are in the range ascribed to small exosomes; (B) Heat map shows that RANK was only detected in CD9-captured EVs from osteoclast-precursors and osteoclasts. Samples were diluted 10X or 2X. Numbers are particle count × 10^3^; (C) EVs captured by anti-CD9 antibody from 2X diluted sample and detected by interferometry (Total), anti-RANK antibody, anti-CD81 antibody, or anti-CD9 antibody. Note that not every EV captured by anti-CD9 antibody is expected to be detected by a second anti-CD9 antibody as antigen may be patched on the capture side of EVs; (D) Very few EVs were captured by CD9 control antibody; (E) EVs captured by Anti-CD81 from 2X diluted samples. CD81 EVs are more common in 4T1 cells than osteoclasts or their precursors, and regardless of cell source, RANK was not detected on EVs captured with an anti-CD81 antibody; (F) Very few EVs were captured by CD81 control antibody. The asterisks in panels C and E indicate significant differences from controls; the *n* = 3. The results indicate statistics performed first by ANOVA, then by *t*-tests, *P* < 0.05; *: *P* < 0.05.

To study RANK-EVs, EVs containing CD9 or CD81 were captured from conditioned media of osteoclasts, osteoclast precursors, or 4T1 cells on SP-IRIS chips carrying anti-CD9, and CD81, or control antibodies. The EVs were then detected, measured by interferometry, and labeled with fluorophore-conjugated antibodies that bind CD9, CD81, or RANK. The measured size of the captured EVs is shown in Figure 3A.

RANK was only detected in EVs from osteoclasts attached to chips through CD9 [Figure 3B]. RANK was detected in 2%-4% of the EVs from osteoclasts captured by the CD9 antibody [Figure 3C]. It was not detected in EVs from pre-osteoclasts. Experimental controls for CD9 using an isotype-matched antibody displayed a very low background [Figure 3D]. RANK was not detected in EVs pulled down by the CD81 antibody [Figure 3E]. The isotype control for CD81 also displayed a very low background [Figure 3F]. As expected, RANK was not detected in EVs from 4T1 cells [Figure 3B, C, and E]. These data suggest RANK is enriched in EVs from osteoclasts that contain CD9.

### CD9/CD81 immunomagnetic isolations

**Figure 4:**
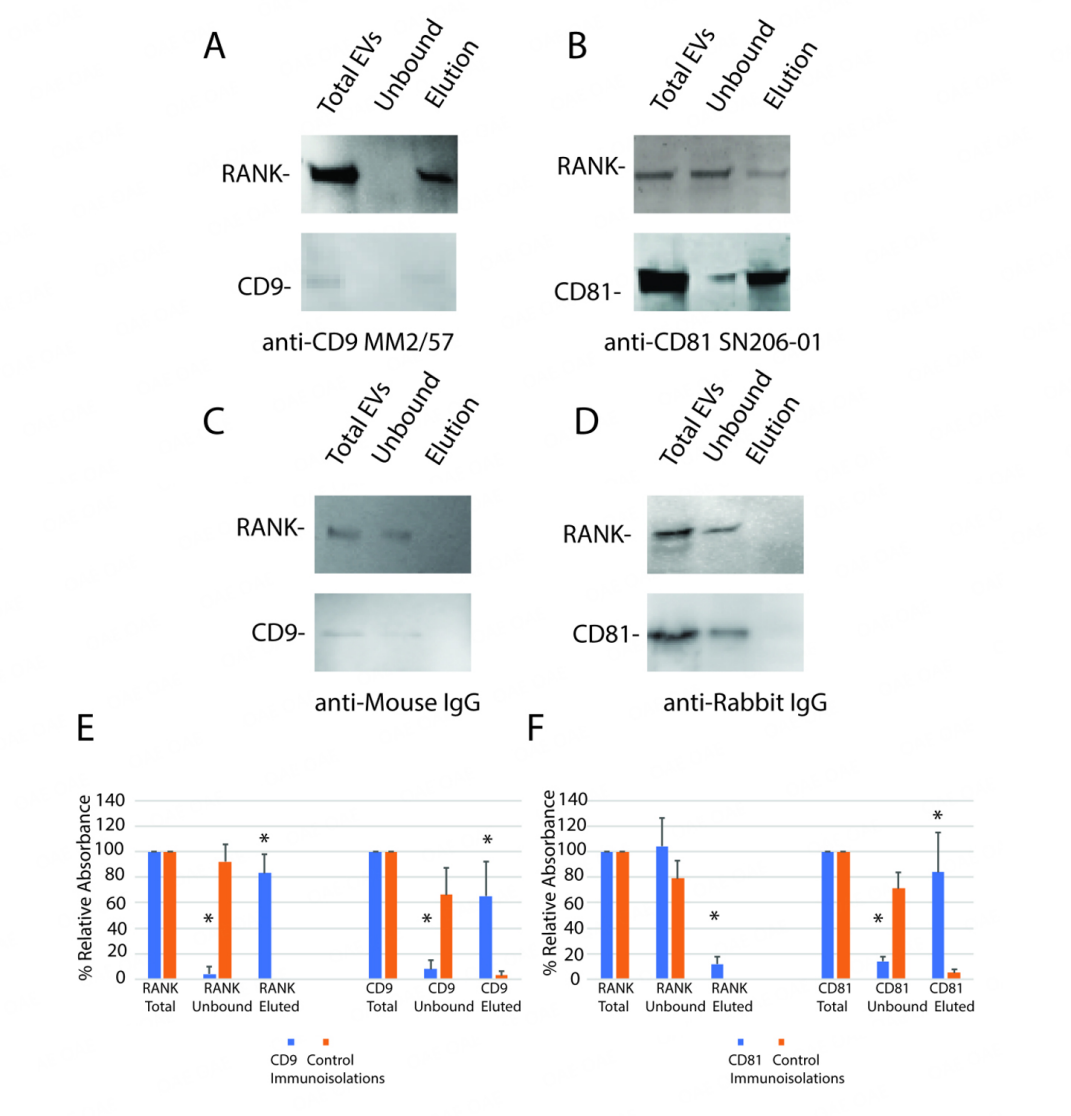
Immunomagnetic bead isolation confirms that RANK is mostly in EVs containing CD9. Conditioned media from osteoclasts from two wells of a 6-well plate for each trial were centrifuged at 5,000 × *g* for 15 min to remove cells and large debris. The conditioned media was subjected to magnetic immunoaffinity isolation. The total conditioned media, the unbound material and bound material eluted with low pH were subjected to SDS-PAGE, blotted to Immobilon P, and probed with antibodies against RANK, CD9 or CD81 as indicated. (A) Affinity isolation with anti-CD9 shows most of RANK was isolated with CD9-containing EVs; (B) Control shows neither RANK nor CD9 was isolated with control antibody; (C) Little RANK was isolated in CD81 EVs; (D) Little binding of RANK or CD81 was detected using a control antibody for anti-CD81; (E) Blots (*n* = 3) of CD9 immunomagnetic isolations were quantitated by densitometry. Most RANK and CD9 were isolated with anti-CD9 immunomagnetic isolation; (F) Blots (*n* = 3) of CD81 immunomagnetic isolations were quantitated by densitometry. A small but significant amount of RANK was detected associated with CD81. Note that the anti-CD81 antibody worked much better for the detection of its target protein in Westerns than the anti-CD9 antibody. Asterisks indicate a difference from control value; *P* < 0.05 by *t*-test; *: *P* < 0.05.

SP-IRIS detected RANK in CD9-positive EVs from pre-osteoclasts and osteoclasts, and not in CD81-positive EVs from osteoclasts. To confirm this result, we performed immunomagnetic bead pull downs from conditioned media of osteoclasts using either immunomagnetic beads linked to a CD9 antibody, a CD81 antibody, or control antibodies. RANK was detected in the CD9 pull down and the conditioned media was mostly cleared of RANK [Figure 4A].

In the anti-CD81 pull down, the majority of RANK was unbound, but some RANK eluted, with CD81. This is consistent with a small fraction of CD81 EVs also containing RANK, even though we could not detect this binding in the SP-IRIS assay [Figure 4B]. Little RANK or CD9 were eluted when a species-specific control antibody was used in place of the anti-CD9 antibody [Figure 4C]. As with the CD9 pull down, little RANK or CD81 were detected in the elution when the control antibody was used in place of the anti-CD81 antibody [Figure 4D]. Results are quantitated by densitometry of blots in Figure 4E (anti-CD9) and Figure 4F (anti-CD81).

## DISCUSSION

For the first time, we show that RANK is selectively enriched in CD9-positive EVs released by osteoclasts. RANK was found by SP-IRIS in 2%-4% of the EVs from osteoclasts that contain CD9. RANK was not detected by SP-IRIS on CD81 carrying EVs. Immunomagnetic isolation using an anti-CD9 antibody pulled down most of both the CD9 and RANK from conditioned media of osteoclasts. A small amount of RANK was isolated with a CD81 antibody by immunomagnetic isolation. These results suggest that osteoclasts have a mechanism for enriching specific subsets of EVs with RANK. The results of this study are consistent with quantitative immunogold labeling in our previous study, which found that 1 in 36 total EVs contained RANK^[8]^. We showed the RANK-EVs could block forward RANKL to RANK signaling like osteoprotegerin^[8]^. Ikebuchi *et al.* later showed that RANK-EVs stimulated RANKL reverse signaling on osteoblasts^[12]^.

RANK is a trimer in cells and RANK molecules would presumably incorporate into EVs as trimers. This idea was recently directly tested in a study that showed RANK in EVs from macrophages binds RANKL on membranes with an affinity (K_D_ = 1-10 nanomolar) that is close to that displayed in cell-cell interactions mediated by RANK and RANKL^[16]^. Since RANK monomers will not bind RANKL^[15]^, this shows that functional RANK trimers are incorporated into EVs, from macrophages. The results are consistent with previous functional assays by our group^[8]^ and by others^[12]^, which showed RANK binding to RANKL triggered changes in cellular differentiation and activity of osteoclasts and osteoblasts. The data from immunogold labeling indicates that the EVs that carry RANK may contain several RANK molecules, most likely trimers, dispersed over the EV. Together with the high binding affinity of the RANK trimer with RANKL trimers, this would make RANK-EVs potent binders of RANKL. If the EVs contain multiple RANK trimers, it may account for the clustering of RANKL on osteoblasts proposed to be crucial for stimulating RANKL reverse signaling and bone formation^[29]^. Further studies will be required to confirm these ideas.

Previous studies have shown CD9 is incorporated into EVs either at the plasma membrane in microvesicles, or in exosomes formed near the plasma membrane in other cell types^[30]^. Localization of CD9 in osteoclasts, and in particular unfused osteoclasts, supports a similar situation. As has been reported previously^[26]^, we found that CD9 is localized near the plasma membrane in unfused osteoclasts and osteoclasts. In contrast, CD81 was detected mostly in cytosolic vesicular compartments. Our results are consistent with RANK-EVs being associated with an EV packaging pathway associated with the plasma membrane, with one possibility being that these are microvesicles.

As osteoclasts form, the percentage of EVs that contain CD9 increases, as does the amount of CD9 in osteoclasts. This is consistent with the overall increase in the expression of CD9 during osteoclastogenesis reported previously^[26]^. Prior studies have also shown that increasing amounts of CD9 are associated with lipid raft domains when osteoclasts are stimulated with RANKL^[26]^. CD9 also plays an important role in the fusion of osteoclast precursors to form multinucleated, mature osteoclasts, and is abundant in active osteoclasts *in vivo*^[26,27,31]^. The increased packaging of RANK into CD9-EVs may reflect increased expression of both RANK^[8]^ and CD9 as osteoclasts mature.

Is it possible that RANK is released in microvesicles shed from the plasma membrane surface? This would be consistent with a previous study examining the location of CD9-EV formation, which concluded that the EVs were formed at the plasma membrane or near the cell’s surface^[30]^. Inconsistent with this idea is the small size of the RANK-containing EVs. Microvesicles are often described as 100 nm in diameter or greater^[32]^, while the RANK-containing EVs measured by electron microscopy were about 50 nm in diameter^[8]^. In addition, both CD9 and CD81 EVs were measured by SP-IRIS to be in the 50-80 nm range, also consistent with exosomes. Finally, images that appeared to capture CD9 and RANK being packaged into the same exosomes in osteoclasts were presented^[12]^. However, better data, perhaps by direct live visualization of RANK-EV release, will be required to resolve this question.

Both CD9 and CD81 have been functionally implicated in osteoclast differentiation and function^[26-28,33]^. They have been suggested to play a role in the fusion of osteoclast precursors to form the mature multinuclear osteoclasts, but there are significant differences in the reports. One study suggested CD9 and CD81 inhibited cell fusion^[28]^, while another argues that CD9 was required for cell fusion^[26]^. Finally, it was pointed out that osteoclasts express a variety of tetraspanins, including CD9 and CD81, and several other proteins, and these may work together in complex ways to support cell fusion^[33]^. Of the twelve tetraspanins identified, only CD82 was detected in osteoclast EVs by mass spectrometry^[24]^. Lipid raft formation triggered by increased CD9 is described as an important consequence of stimulation of osteoclasts through the RANKL/RANK interaction^[26]^. Such rafts may serve as platforms for new signaling pathways^[34]^. They may also serve in the formation of EVs^[34,35]^. This provides a potential mechanistic basis that is testable for links between CD9 and RANK in EVs.

This study is limited by the small number of proteins examined and by the use of only two techniques, SP-IRIS and immunomagnetic isolation. The use of more markers and other techniques, such as ZetaView (Particle Matrix), which combines nanoparticle tracking with fluorescent labeling, will likely lead to a more detailed understanding of the subpopulations of EVs released by osteoclasts, including the subpopulation containing RANK. This study does not address the mechanism of the association between RANK and CD9 but does generate testable questions regarding whether RANK-EV are microvesicles or exosomes, and whether RANK-EV generation is linked to lipid rafts. Finally, this study is in mice. Although mice have proven a useful model for bone remodeling, future studies using human cells would be useful.

In conclusion, for the first time, we show that RANK is enriched in a subpopulation of CD9-containing EVs. This suggests that the packaging of RANK into EVs occurs along a distinct EV production pathway. Currently, the underlying mechanisms for selectively packaging of RANK or CD9 into EVs are not known. However, as this pathway is elucidated, means to stimulate the pathway and enhance the packaging of RANK into osteoclast EVs may be identified. Therapeutic stimulation of the number of RANK-containing EVs released by osteoclasts provides an attractive approach to treating osteoporosis. Because RANK-containing EVs simultaneously inhibit bone resorption and stimulate bone formation, increasing their production locally would be expected to be a potent means to therapeutically increase bone density.

## DECLARATIONS

### Availability of data and materials

Data and materials supporting the published data will be shared on reasonable requests.

### Copyright

© The Author(s) 2023.
